# 2-(4-Bromo­benzene­sulfonamido)acetic acid

**DOI:** 10.1107/S160053680902604X

**Published:** 2009-07-11

**Authors:** Muhammad Nadeem Arshad, Islam Ullah Khan, Muhammad Shafiq, Muhammad Naeem Khan, Helen Stoeckli-Evans

**Affiliations:** a Materials Chemistry Laboratory, Department of Chemistry, GC University, Lahore, Pakistan; b PCSIR, Laboratories Complex, Ferozpur Road, Lahore, Pakistan; c Institute of Physics, Université of Neuchâtel, Rue Emile-Argand 11, CH-2009 Neuchâtel, Switzerland

## Abstract

The title compound, C_8_H_8_BrNO_4_S, a halogenated sulfon­amide, was prepared by basic hydrolysis of the methyl ester. In the crystal, mol­ecules form centrosymmetric hydrogen-bonded dimers *via* the carboxyl groups. These dimers are further linked by N—H⋯O inter­actions involving the carbonyl O and amide H atoms, forming a ribbon-like structure propagating in [010]. These ribbons are further linked *via* C—H⋯O inter­actions, forming a three-dimensional network.

## Related literature

For details of the crystal structure of the methyl ester of the title compound, see: Arshad *et al.* (2008*b* ▶). For related structures, see: Arshad *et al.* (2008*a* ▶); Arshad *et al.* (2009 ▶). For related thia­zine heterocycles, see: Arshad *et al.* (2008*c* ▶). For hydrogen-bonding patterns, see: Bernstein *et al.* (1995 ▶).
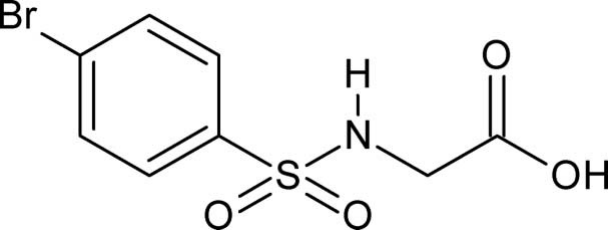


## Experimental

### 

#### Crystal data

C_8_H_8_BrNO_4_S
*M* *_r_* = 294.12
Triclinic, 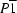

*a* = 5.0042 (4) Å
*b* = 7.9997 (6) Å
*c* = 13.2289 (11) Å
α = 79.691 (4)°
β = 88.667 (5)°
γ = 81.404 (4)°
*V* = 515.18 (7) Å^3^
*Z* = 2
Mo *K*α radiation
μ = 4.18 mm^−1^
*T* = 296 K
0.28 × 0.17 × 0.11 mm

#### Data collection

Bruker Kappa APEXII CCD diffractometer
Absorption correction: multi-scan (*SADABS*; Bruker, 2007 ▶) *T*_min_ = 0.612, *T*_max_ = 0.632
10359 measured reflections
2557 independent reflections
1243 reflections with *I* > 2σ(*I*)
*R*_int_ = 0.056

#### Refinement

*R*[*F* ^2^ > 2σ(*F* ^2^)] = 0.058
*wR*(*F* ^2^) = 0.168
*S* = 0.95
2557 reflections
137 parameters
H-atom parameters constrained
Δρ_max_ = 1.15 e Å^−3^
Δρ_min_ = −0.38 e Å^−3^

### 

Data collection: *APEX2* (Bruker, 2007 ▶); cell refinement: *SAINT* (Bruker, 2007 ▶); data reduction: *SAINT*; program(s) used to solve structure: *SHELXS97* (Sheldrick, 2008 ▶); program(s) used to refine structure: *SHELXL97* (Sheldrick, 2008 ▶); molecular graphics: *PLATON* (Spek, 2009 ▶) and *Mercury* (Macrae *et al.*, 2006 ▶); software used to prepare material for publication: *SHELXL97*.

## Supplementary Material

Crystal structure: contains datablocks I, global. DOI: 10.1107/S160053680902604X/tk2493sup1.cif

Structure factors: contains datablocks I. DOI: 10.1107/S160053680902604X/tk2493Isup2.hkl

Additional supplementary materials:  crystallographic information; 3D view; checkCIF report

## Figures and Tables

**Table 1:** Hydrogen-bond geometry (Å, °)

*D*—H⋯*A*	*D*—H	H⋯*A*	*D*⋯*A*	*D*—H⋯*A*
O3—H3*O*⋯O4^i^	0.82	1.85	2.671 (5)	174
N1—H1⋯O4^ii^	0.86	2.38	3.124 (5)	146
C2—H2⋯O1^iii^	0.93	2.53	3.384 (7)	153
C3—H3⋯O3^iv^	0.93	2.50	3.423 (7)	170

Symmetry codes: (i) 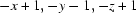; (ii) 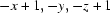; (iii) 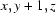; (iv) 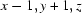.

**Fig. 1.**
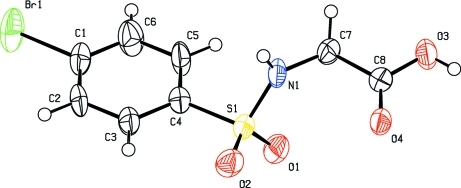
The molecular structure of (I), with displacement ellipsoids drawn at the 50% probability level.

**Fig. 2.**
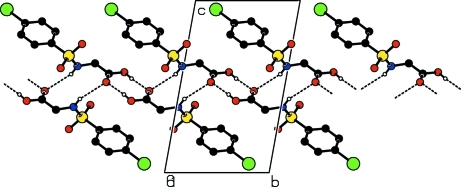
A view along the a axis of the crystal packing of compound (I), with O—H···O and N—H···O hydrogen bonds drawn as dashed lines [see Table 1 for details; H atoms not involved in hydrogen bonding have been omitted for clarity].

**Fig. 3.**
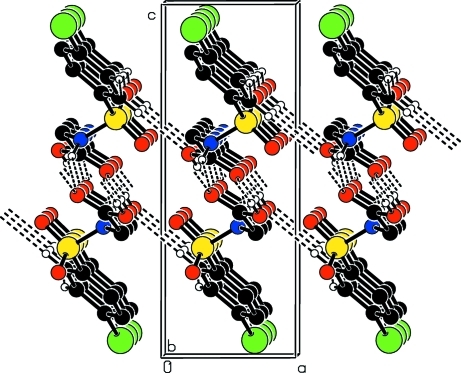
A view along the b axis of the crystal packing of (I), with O—H···O, N—H···O and C—H···O hydrogen bonds drawn as dashed lines [see Table 1 for details; H atoms not involved in hydrogen bonding have been omitted for clarity].

**Fig. 4.**
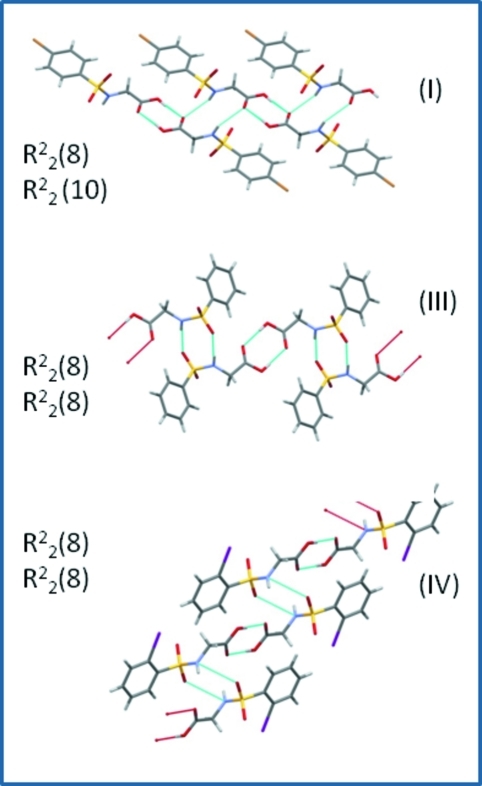
A view of the hydrogen bonding patterns in the three acid compounds: (I), (III) and (IV). The hydrogen bonds are shown as pale-blue lines.

## supplementary crystallographic information

### Comment

The title compound, (I), was prepared by basic hydrolysis of methyl (4-bromobenzenesulfonamido)acetate (II) (Arshad *et al.*, 2008*b*), in a continuation of our studies on the synthesis of thiazine related heterocycles (Arshad *et al.*, 2008*c*). We have previously reported the crystal structures of 2-(benzenesulfonamido)acetic acid (III) (Arshad *et al.*, 2008*a*) and 2-(2-iodobenzenesulfonamido)acetic acid (IV) (Arshad *et al.*, 2009).

The molecular structure of (I), Fig. 1, reveals the bond lengths and angles are similar to those found for compounds (II), (III) and (IV).

The presence of the carboxylic acid group leads to the formation of characteristic O—H···O hydrogen-bonded centrosymmetric dimers (Table 1 and Fig. 2). These dimers are linked *via* N1—H1···O4 interactions, involving the carbonyl O-atom and the H-atom of the amido group, to form a ribbon-like structure propagating in the [010] direction (Table 1 and Fig. 2). The ribbons are further linked by C—H···O interactions to form a 3-D network (Table 1 and Fig. 3).

It is interesting to compare the hydrogen bonding patterns in the three acids; (I), (III) and (IV). The formation of the hydrogen bonded carboxylic acid dimers is the same in all three compounds, i.e. *R*^2^_2_(8) (Bernstein *et al.*, 1995). The N—H···O hydrogen-bonding involves the sulfonamido groups in (III) and (IV) [*R*^2^_2_(8)], while in (I) it involves the carbonyl O-atom (O4) and the H-atom of the amido group (Table 1). This leads to a larger hydrogen-bonded ring of the form [*R*^2^_2_(10)], as shown in Fig. 4.

### Experimental

Methyl (4-bromobenzenesulfonamido)acetate(II) (Arshad *et al.*, 2008*b*) (1.0 g, 3.247 mmol) was dissolved in an aqueous sodium hydroxide solution (10%, 10 ml). The resulting solution was refluxed for an hour. The reaction mixture was then cooled to room temperature and acidified with 1 N HCl. A white precipitate was obtained. This was filtered off, washed with distilled water and dried. Crystals were obtained by recrystallization from methanol.

### Refinement

The H-atoms were included in calculated positions and treated as riding atoms: O—H = 0.82 Å, N—H = 0.86 Å, C—H = 0.93 - 0.97 Å, with *U*_iso_(H) = k × *U*_eq_(parent O–, N– or C-atom), where k = 1.5 for OH, and 1.2 for N- and C-bound H-atoms.

The maximum and minimum residual electron density peaks of 1.15 and -0.38 eÅ^-3^, respectively, were located at 1.10 Å and 0.78 Å, respectively, from atom Br1.

### Crystal data

**Table:**

C_8_H_8_BrNO_4_S	*Z* = 2
*M**_r_* = 294.12	*F*(000) = 292
Triclinic, *P*1	*D*_x_ = 1.896 Mg m^−^^3^
Hall symbol: -P 1	Mo *K*α radiation, λ = 0.71073 Å
*a* = 5.0042 (4) Å	Cell parameters from 1869 reflections
*b* = 7.9997 (6) Å	θ = 2.2–21.8°
*c* = 13.2289 (11) Å	µ = 4.18 mm^−^^1^
α = 79.691 (4)°	*T* = 296 K
β = 88.667 (5)°	Needle, colorless
γ = 81.404 (4)°	0.28 × 0.17 × 0.11 mm
*V* = 515.18 (7) Å^3^	

### Data collection

**Table:**

Bruker Kappa APEXII CCD diffractometer	2557 independent reflections
Radiation source: fine-focus sealed tube	1243 reflections with *I* > 2σ(*I*)
graphite	*R*_int_ = 0.056
φ and ω scans	θ_max_ = 28.3°, θ_min_ = 2.6°
Absorption correction: multi-scan (*SADABS*; Bruker, 2007)	*h* = −6→6
*T*_min_ = 0.612, *T*_max_ = 0.632	*k* = −10→10
10359 measured reflections	*l* = −17→17

### Refinement

**Table:**

Refinement on *F*^2^	Primary atom site location: structure-invariant direct methods
Least-squares matrix: full	Secondary atom site location: difference Fourier map
*R*[*F*^2^ > 2σ(*F*^2^)] = 0.058	Hydrogen site location: inferred from neighbouring sites
*wR*(*F*^2^) = 0.168	H-atom parameters constrained
*S* = 0.95	*w* = 1/[σ^2^(*F*_o_^2^) + (0.0872*P*)^2^ + 0.2177*P*] where *P* = (*F*_o_^2^ + 2*F*_c_^2^)/3
2557 reflections	(Δ/σ)_max_ < 0.001
137 parameters	Δρ_max_ = 1.15 e Å^−^^3^
0 restraints	Δρ_min_ = −0.38 e Å^−^^3^

### Special details

**Table:**

Geometry. All e.s.d.'s (except the e.s.d. in the dihedral angle between two l.s. planes) are estimated using the full covariance matrix. The cell e.s.d.'s are taken into account individually in the estimation of e.s.d.'s in distances, angles and torsion angles; correlations between e.s.d.'s in cell parameters are only used when they are defined by crystal symmetry. An approximate (isotropic) treatment of cell e.s.d.'s is used for estimating e.s.d.'s involving l.s. planes.
Refinement. Refinement of *F*^2^ against ALL reflections. The weighted *R*-factor *wR* and goodness of fit *S* are based on *F*^2^, conventional *R*-factors *R* are based on *F*, with *F* set to zero for negative *F*^2^. The threshold expression of *F*^2^ > σ(*F*^2^) is used only for calculating *R*-factors(gt) *etc*. and is not relevant to the choice of reflections for refinement. *R*-factors based on *F*^2^ are statistically about twice as large as those based on *F*, and *R*- factors based on ALL data will be even larger.

### Fractional atomic coordinates and isotropic or equivalent isotropic displacement parameters (Å^2^)

**Table:**

	*x*	*y*	*z*	*U*_iso_*/*U*_eq_	
Br1	0.70371 (17)	0.78972 (8)	0.05167 (5)	0.0774 (4)	
S1	0.3354 (2)	0.11701 (17)	0.32101 (10)	0.0365 (4)	
O1	0.2650 (7)	0.0106 (5)	0.2528 (3)	0.0486 (10)	
O2	0.1387 (7)	0.1738 (5)	0.3930 (3)	0.0492 (10)	
O3	0.7775 (7)	−0.4424 (5)	0.4169 (3)	0.0505 (10)	
H3O	0.7174	−0.5221	0.4536	0.076*	
O4	0.4156 (7)	−0.2871 (4)	0.4743 (3)	0.0427 (9)	
N1	0.5998 (8)	0.0162 (5)	0.3816 (3)	0.0389 (10)	
H1	0.6632	0.0630	0.4278	0.047*	
C1	0.5927 (12)	0.5917 (7)	0.1309 (4)	0.0456 (14)	
C2	0.3808 (12)	0.6089 (7)	0.1979 (5)	0.0495 (15)	
H2	0.2914	0.7172	0.2039	0.059*	
C3	0.3022 (11)	0.4635 (7)	0.2560 (5)	0.0453 (14)	
H3	0.1575	0.4737	0.3010	0.054*	
C4	0.4368 (10)	0.3039 (7)	0.2478 (4)	0.0355 (12)	
C5	0.6490 (11)	0.2867 (7)	0.1801 (4)	0.0489 (15)	
H5	0.7386	0.1784	0.1742	0.059*	
C6	0.7271 (13)	0.4320 (8)	0.1211 (5)	0.0584 (17)	
H6	0.8695	0.4221	0.0751	0.070*	
C7	0.7389 (10)	−0.1470 (6)	0.3652 (4)	0.0410 (13)	
H7A	0.9274	−0.1559	0.3839	0.049*	
H7B	0.7316	−0.1519	0.2925	0.049*	
C8	0.6253 (10)	−0.2978 (7)	0.4249 (4)	0.0368 (12)	

### Atomic displacement parameters (Å^2^)

**Table:**

	*U*^11^	*U*^22^	*U*^33^	*U*^12^	*U*^13^	*U*^23^
Br1	0.1144 (7)	0.0372 (4)	0.0728 (6)	−0.0176 (4)	0.0098 (4)	0.0154 (3)
S1	0.0376 (7)	0.0264 (7)	0.0458 (8)	−0.0085 (5)	0.0029 (5)	−0.0043 (6)
O1	0.053 (2)	0.037 (2)	0.060 (3)	−0.0124 (18)	−0.0073 (18)	−0.0143 (19)
O2	0.045 (2)	0.046 (2)	0.058 (2)	−0.0118 (18)	0.0163 (18)	−0.011 (2)
O3	0.050 (2)	0.027 (2)	0.069 (3)	0.0001 (18)	0.0167 (19)	−0.001 (2)
O4	0.0398 (19)	0.027 (2)	0.059 (2)	−0.0058 (16)	0.0127 (17)	−0.0008 (17)
N1	0.050 (2)	0.019 (2)	0.046 (3)	−0.0062 (19)	−0.002 (2)	−0.001 (2)
C1	0.064 (4)	0.026 (3)	0.044 (3)	−0.009 (3)	−0.004 (3)	0.002 (3)
C2	0.059 (3)	0.017 (3)	0.068 (4)	0.003 (3)	0.000 (3)	−0.006 (3)
C3	0.045 (3)	0.025 (3)	0.065 (4)	−0.002 (2)	0.008 (3)	−0.007 (3)
C4	0.041 (3)	0.027 (3)	0.038 (3)	−0.003 (2)	−0.001 (2)	−0.005 (2)
C5	0.064 (4)	0.022 (3)	0.054 (4)	0.001 (3)	0.013 (3)	0.004 (3)
C6	0.073 (4)	0.040 (4)	0.054 (4)	−0.001 (3)	0.021 (3)	0.006 (3)
C7	0.039 (3)	0.032 (3)	0.049 (3)	−0.010 (2)	0.009 (2)	0.001 (3)
C8	0.035 (3)	0.033 (3)	0.042 (3)	−0.007 (2)	0.000 (2)	−0.003 (2)

### Geometric parameters (Å, °)

**Table:**

Br1—C1	1.886 (5)	C2—C3	1.379 (7)
S1—O1	1.429 (4)	C2—H2	0.9300
S1—O2	1.436 (4)	C3—C4	1.374 (7)
S1—N1	1.594 (4)	C3—H3	0.9300
S1—C4	1.765 (5)	C4—C5	1.380 (7)
O3—C8	1.306 (6)	C5—C6	1.382 (7)
O3—H3O	0.8200	C5—H5	0.9300
O4—C8	1.223 (6)	C6—H6	0.9300
N1—C7	1.436 (6)	C7—C8	1.499 (6)
N1—H1	0.8600	C7—H7A	0.9700
C1—C2	1.373 (8)	C7—H7B	0.9700
C1—C6	1.379 (8)		
			
O1—S1—O2	119.3 (2)	C3—C4—C5	120.5 (5)
O1—S1—N1	106.7 (2)	C3—C4—S1	120.6 (4)
O2—S1—N1	109.3 (2)	C5—C4—S1	118.9 (4)
O1—S1—C4	108.9 (2)	C4—C5—C6	119.4 (5)
O2—S1—C4	106.5 (2)	C4—C5—H5	120.3
N1—S1—C4	105.3 (2)	C6—C5—H5	120.3
C8—O3—H3O	109.5	C1—C6—C5	119.6 (5)
C7—N1—S1	124.8 (4)	C1—C6—H6	120.2
C7—N1—H1	117.6	C5—C6—H6	120.2
S1—N1—H1	117.5	N1—C7—C8	113.8 (4)
C2—C1—C6	121.0 (5)	N1—C7—H7A	108.8
C2—C1—Br1	119.6 (4)	C8—C7—H7A	108.8
C6—C1—Br1	119.4 (4)	N1—C7—H7B	108.8
C1—C2—C3	119.2 (5)	C8—C7—H7B	108.8
C1—C2—H2	120.4	H7A—C7—H7B	107.7
C3—C2—H2	120.4	O4—C8—O3	124.3 (5)
C4—C3—C2	120.2 (5)	O4—C8—C7	124.4 (5)
C4—C3—H3	119.9	O3—C8—C7	111.4 (4)
C2—C3—H3	119.9		
			
O1—S1—N1—C7	2.2 (4)	O1—S1—C4—C5	−57.6 (5)
O2—S1—N1—C7	132.5 (4)	O2—S1—C4—C5	172.6 (4)
C4—S1—N1—C7	−113.5 (4)	N1—S1—C4—C5	56.6 (5)
C6—C1—C2—C3	0.0 (9)	C3—C4—C5—C6	0.6 (9)
Br1—C1—C2—C3	−179.5 (4)	S1—C4—C5—C6	178.9 (5)
C1—C2—C3—C4	0.8 (9)	C2—C1—C6—C5	−0.5 (10)
C2—C3—C4—C5	−1.1 (8)	Br1—C1—C6—C5	179.1 (5)
C2—C3—C4—S1	−179.4 (4)	C4—C5—C6—C1	0.2 (9)
O1—S1—C4—C3	120.7 (5)	S1—N1—C7—C8	−85.5 (5)
O2—S1—C4—C3	−9.1 (5)	N1—C7—C8—O4	8.2 (8)
N1—S1—C4—C3	−125.2 (5)	N1—C7—C8—O3	−172.4 (4)

### Hydrogen-bond geometry (Å, °)

**Table:**

*D*—H···*A*	*D*—H	H···*A*	*D*···*A*	*D*—H···*A*
O3—H3O···O4^i^	0.82	1.85	2.671 (5)	174
N1—H1···O4^ii^	0.86	2.38	3.124 (5)	146
C2—H2···O1^iii^	0.93	2.53	3.384 (7)	153
C3—H3···O3^iv^	0.93	2.50	3.423 (7)	170
C3—H3···O2	0.93	2.50	2.884 (7)	105

Symmetry codes: (i) −*x*+1, −*y*−1, −*z*+1; (ii) −*x*+1, −*y*, −*z*+1; (iii) *x*, *y*+1, *z*; (iv) *x*−1, *y*+1, *z*.
